# 592 Strategy for Improving Wound Care Compliance in the Outpatient Setting by using Take-home Visual Aids

**DOI:** 10.1093/jbcr/irac012.220

**Published:** 2022-03-23

**Authors:** David G'sell, Sydney Smith, Carl A Flores, Abbey Peterson, Lacy Virgadamo, Herb A Phelan, Nicole M Kopari, Jeffrey E Carter

**Affiliations:** University Medical Center New Orleans, New Orleans, Louisiana; University Medical Center, New Orleans, Louisiana; University Medical Center New Orleans, New Orleans, Louisiana; University Medical Center, Metairie, Louisiana; UMC Burn Center, New Orleans, Louisiana; LSUHSC-New Orleans Department of Surgery, New Orleans, Louisiana; Children's Hospital New Orleans, New Orleans, Louisiana; University Medical Center- New Orleans, New Orleans, Louisiana

## Abstract

**Introduction:**

Wound care compliance is a critical component in the success of treating burns in the outpatient setting. Patients and caregivers are educated with demonstration and written materials which have demonstrated a 24-hour retention rate of 30% and 10% respectively. This can leave the patient at higher risk for infection, increased pain from suboptimal dressings, and feelings of frustration. Research has shown that visual learners make up 65% of the population, with auditory learners at 30%, and tactile learners at 5%. We assessed that a combination of demonstration and visual aids could better assist different learning styles and improve wound care compliance. Our study goal was to assess the efficacy of the visual aids component through new patient encounters in the emergency department and outpatient setting using a six-question survey at subsequent encounters.

**Methods:**

The study design is a prospective analysis with comparison to historical controls. Visual aids were designed by the burn physician assistants with assistance at an ABA-verified burn center. We created four double-sided cards made out of a water-resistant synthetic paper, with one for each of our most used dressings. The content of the cards included one of the following: bacitracin/fine mesh gauze with bismuth tribromophenate, bacitracin with low-adherent acetate gauze, silver nylon dressings, and silver foam dressings. Each card contains moulage wounds, step-by-step, and corresponding written instructions at a 4^th^ grade education reading level. These visual aids were given to patients being discharged from the emergency department, or to new patients in the burn clinic. A six-question survey was administered at one week follow-up encounters with a scale of 1-10 (one being least helpful, and ten being the most helpful) assessing patients understanding of burn wound care and compliance. Compliance rates were abstracted from historical controls with similar burn wound severity.

**Results:**

Limited data is available at the time of submission as the study is currently in-progress and anticipated to be completed by March 2021. We will be using descriptive statistics and comparative analysis to evaluate the results.

**Conclusions:**

Patients initial feedback has been overall positive with a corresponding compliance rate that is successful. Our patients verbalized their approval, with multiple patients stating that they plan to keep the wound care card for any burn injuries that might occur in the future. Additional research is needed to examine the impact of combined demonstration, tactile, and auditory learning aids. In addition, we plan to further expand our engagement effort to include similar wound care cards for pediatric patients as well as language alternative cards to meet our surrounding community's needs.

